# Comparative overall survival analysis of chordomas of the base of the skull from the Surveillance, Epidemiology, and End Results (SEER) program between 2000 and 2020

**DOI:** 10.1007/s10143-024-02815-0

**Published:** 2024-09-25

**Authors:** Kevin E. Agner, Michael C. Larkins

**Affiliations:** 1 The Ohio State University College of Medicine, 370 W. 9Th Avenue, Columbus, OH 43210 USA; 2 East Carolina University Brody School of Medicine, 600 Moye Blvd, Greenville, NC 27834 USA; 3 Department of Emergency Medicine, Boonshoft School of Medicine at Wright State University, 2555 University Blvd, Fairborn, OH USA

**Keywords:** Chordoma, Skull base tumors, Surgical oncology, Survival analysis

## Abstract

Chordomas of the skull base are rare, slow growing, locally invasive cancers with limited long-term survival analysis reported in the literature. We seek to provide comparative survival analysis of patients on a long-term (20-year) basis using population-level data. The Surveillance, Epidemiology, and End Results (SEER) program was queried for cases of chordoma relegated to the base of the skull, diagnosed between 2000 and 2020. Demographic, disease, and treatment information were analyzed using Cox proportional hazards and log-rank comparisons. 630 patients with chordoma of the skull base were identified. Age ≤ 49 years at diagnosis was associated with increased five-, 10-, and 20-year overall survival (hazard ratio (HR) = 0.39, 0.33, and 0.30, respectively; *p* < 0.001 for all). Treatment with surgery and adjuvant radiotherapy was associated with increased five-, 10-, and 20-year survival (HR = 0.71, 0.79, and 0.79, respectively; *p* < 0.001 for all). On univariate analysis, widowed patients had decreased survival (20-year overall survival = 34.8% [15.3%-34.8%] compared to married patients (74.4% [68.1%-80.8%]. Surgery remains the primary treatment associated with increased survival among patients with chordoma of the skull base, with adjuvant radiotherapy serving a complimentary role. Demographic factors such as marital status are also associated with changes in survival.

**Supplementary Information:**

The online version contains supplementary material available at 10.1007/s10143-024-02815-0.

## Introduction

Skull base chordomas (SBC) are rare bone tumors originating from embryonic remnants of the notochord, primarily affecting the base of the skull, spine, and tailbone region [7]. Despite commonly presenting as benign tumors, SBC exhibit locally aggressive behavior and grow slowly, posing significant challenges for effective clinical management. These tumors represent a small fraction of cancers, accounting for 1–4% of all primary bone tumors, with an incidence rate of approximately 8 per 10 million individuals.

Treatment for SBC remains complex and individualized, though primarily surgical management is most typically reported in the literature [1, 5, 14]. The extent of tumor removal has an impact on patient outcomes, with the literature demonstrating improved survival and reduced recurrence rates associated with complete surgical removal, particularly for skull base and sacral chordomas [2, 10, 14]. However, achieving complete resection can be difficult due to the tumor's proximity to critical nerves and blood vessels, especially in the skull base region. This necessitates the use of specialized surgical approaches depending on tumor location and size [1, 6]. Radiotherapy (RT) serves as a valuable complementary treatment, particularly for partially removed or inoperable tumors [3, 13]. Proton therapy and intensity-modulated RT allow for the delivery of higher, more precise radiation doses, potentially enhancing local control and overall survival [5].

A SEER database analysis by Chambers et al. reported an overall three-, five-, and 10-year survival for cranial chordomas to be 80.9%, 73.5%, and 58.7% respectively [3]. Another SBC SEER analysis identified several factors influencing survival, including age at diagnosis, tumor location, disease stage, surgical treatment, and tumor size [10]. While complete surgical removal did not significantly impact overall survival compared to partial removal, it did demonstrate a positive effect on progression-free survival.

To date, no long-term (20-year), comprehensive multivariate analysis of survival in relation to demographic, disease, and treatment characteristics has been performed. This is despite the high survival rates reported among shorter timeframes.

## Methods

### Patient Identification

This retrospective study utilized data queried from the National Cancer Institute's Surveillance, Epidemiology, and End Results (SEER) 17-registry Incidence database, which lists cases diagnosed between the years 2000 and 2020 [15]. Patients diagnosed with SBC were identified using the International Classification of Diseases for Oncology, Third Edition (ICD-O-3) histology codes 9370/3: Chordoma, not otherwise specified (NOS), 9371/3: Chondroid chordoma, and 9372/3: Dedifferentiated chordoma, with the further specification of the primary site as the skull base through the appropriate SEER site recode. Additionally, all cases were required to have a behavior code ICD-O-3 characterized as “Malignant.”

### Case Analysis

Demographic, disease, and treatment variables were collected from the SEER Program and assessed for association with a change in five-, 10-, and 20-year overall survival (OS). Analysis was conducted using SPSS (Version 29.0; Armonk, NY: IBM Corp.), with a p-value of < 0.05 as the cutoff for statistical significance and 95% confidence intervals reported in brackets [95% CI]. Cox proportional hazards regression analysis was used to evaluate multivariate associations with respect to survival, while log-rank tests were used to compare survival curves on univariate analysis. Kaplan–Meier curves were used to visualize comparisons between groups.

## Results

### Cohort Characterization

A total of 630 patients diagnosed with SBC between 2000 and 2020 were identified in the Surveillance, Epidemiology, and End Results (SEER) database. Patient age was reported in five-year intervals, with the median age at diagnosis being 45–49 years. The majority of patients were male (53.2%), White (80.6%), and married (54.6%). Disease stage is reported within the SEER Program as summary stage [16]; the most common summary stage was localized disease (48.4%), followed by regional disease (39.0%), distant disease (5.9%), unknown/unstaged disease (4.6%), and unstaged disease (2.1%). The majority of patients (89.8%) had a histological diagnosis of SBC, not otherwise specified (NOS). Demographic and disease characteristics are summarized in Table 1.

**Table 1:** Demographic and disease characteristics of patients diagnosed with SBC in the US between 2000 and 2020 as identified in the SEER database (NOS: not otherwise specified)

Variable	Number (% of Cohort; n = 630)
*Age at Diagnosis*	
1–29 Years	123 (19.5%)
30–44 Years	151 (23.9%)
45–54 Years	122 (19.3%)
55–69 Years	143 (22.6%)
70–85 + Years	91 (14.4%)
*Age at Diagnosis (Split by Median Age)*	
0–49 Years	331 (52.5%)
50–85 + Years	299 (47.5%)
*Sex*	
Male	335 (53.2%)
Female	295 (46.8%)
*Race*	
American Indian/Alaska Native	3 (0.5%)
Asian/Pacific Islander	82 (13.0%)
Black	31 (4.9%)
White	508 (80.6%)
Unknown	6 (1.0%)
*Marital Status*	
Single	180 (39.8%)
Married	344 (54.6%)
Unknown	25 (4.0%)
*Summary Stage*	
Unstaged/Incomplete Staging	42 (6.7%)
Local	305 (48.4%)
Regional	246 (39.0%)
Distant	37 (5.9%)
*Histological Type*	
9370/3: Chordoma, NOS	566 (89.8%)
9371/3: Chondroid chordoma	61 (9.7%)
9372/3: Dedifferentiated chordoma	3 (0.5%)
*Overall Survival*	
Five-Year Overall Survival	532 (84.4%)
10-Year Overall Survival	480 (76.2%)
20-Year Overall Survival	448 (71.1%)

Information on patient treatment can be found in Table 2. 87.3% of the identified cohort underwent surgical treatment for SBC, while 56.2% received RT. 53.2% underwent both surgery and RT, and of these 99.4% received RT adjuvant to surgical treatment.

**Table 2:** Treatment characteristics of patients diagnosed with chordoma of the skull base (SBC) in the US between 2000 and 2020

Variable	Number (% of Cohort; n = 630)
*Surgery*	
No Surgery	80 (12.7%)
Surgery Performed	550 (87.3%)
*Radiotherapy (RT)*	
No RT/Unknown	276 (43.8%)
RT Administered	354 (56.2%
*RT Sequence*	
No RT and/or no surgery	295 (46.8%)
RT after surgery	333 (52.9%)
RT before Surgery	2 (0.3%)
*Surgical Procedure*	
None	79 (12.5%)
Subtotal Resection	369 (58.6%)
Gross Total Resection	163 (25.9%)
Surgery Not Otherwise Specified/Unknown	19 (3.0%)
*Surgery and Radiation Combinations*	
Neither	61 (9.7%)
Surgery only	215 (34.1%)
RT only	19 (3.0%)
Surgery and RT*	335 (53.2%)
*Chemotherapy*	
Yes	17 (2.7%)
No	613 (97.3%)

*Of these patients, 0.6% had neoadjuvant RT and the remainder had adjuvant RT

Treatment characteristics of patients diagnosed with SBC in the US between 2000 and 2020 as identified in the SEER database. The majority of patients (87.3%) underwent surgery, with subtotal resection being the most common procedure (58.6%). 52.9% received radiation, while only 2.7% of patients received chemotherapy

### Multivariate Analysis

Cox regression analysis was performed to evaluate the impact of various factors on overall survival (OS) at five, 10, and 20 years after diagnosis. The results are summarized in Table 3. Older age at diagnosis (> 49 years compared to ≤ 49 years) was consistently associated with decreased OS at 5, 10, and 20 years (Hazard Ratio (HR) = 2.49, 3.00, and 3.22, respectively; *p* < 0.001 for all). Married patients had increased survival compared to single patients at 5 years (HR = 0.67, p = 0.012); however, at 10 years (p = 0.060) and 20 years (p = 0.082), the same trend was not present. Disease stage was associated with a higher risk of death when comparing regional to local disease, and these findings were significant at the five- and 20-year time periods (5y HR = 1.12, p = 0.044; 10y HR 1.09, p = 0.051, and 20y HR = 1.10, p = 0.016). Treatment with chemotherapy was also associated with decreased survival (5y HR = 3.28, p = 0.007, 10y HR = 3.38, *p* < 0.0001; and 20y HR = 3.12, *p* < 0.001). Treatment with both surgery and RT (whether neoadjuvant or adjuvant) was associated with improved OS at all time points compared to either unimodal or no treatment (5y HR = 0.73, p = 0.003; 10y HR = 0.81, *p* < 0.001; 20y HR = 0.81, p = 0.002). It is important to note that 99.4% of the patients that received both surgery and RT had RT administered in an adjuvant fashion (see Table 2).

**Table 3:** Cox regression analysis of five, 10, and 20-year overall survival among patients with chordoma of the skull base (SBC)

Variable	*p*-value	Hazard Ratio
Five-year Overall Survival		
Age (> 49y vs ≤ 49y)*	< 0.001	2.49
Sex (Male vs Female)	0.55	1.13
Race (White vs American Indian)	0.884	1.01
Marital Status (Married vs Single)	0.012	0.67
Histological Type (ICD-O-3: 9372 vs 9370)	0.58	0.823
Stage (Regional vs Local)	0.044	1.12
Chemotherapy (Y vs N/Unk)	0.007	3.28
Treatment Plan (Both vs None)	< 0.001	0.73
10-year Overall Survival		
Age (> 49y vs ≤ 49y)*	< 0.001	3.00
Sex (Male vs Female)	0.21	1.24
Race (White vs American Indian)	0.75	1.03
Marital Status (Married vs Single)	0.060	0.78
Histological Type (9372 vs 9370)	0.316	0.76
Stage (Regional vs Local)	0.051	1.09
Chemotherapy (Y vs N/Unk)	< . 0001	3.38
Treatment Plan (Both vs None)	0.003	0.81
20-year Overall Survival		
Age (> 49y vs ≤ 49y)*	< 0.001	3.22
Sex (Male vs Female)	0.16	1.24
Race (White vs American Indian)	0.44	1.06
Marital Status (Married vs Single)	0.082	0.810
Histological Type (9372 vs 9370)	0.603	0.89
Stage (Regional vs Local)	0.016	1.10
Chemotherapy (Y vs N/Unk)	< 0.001	3.12
Treatment Plan (Both vs None)	0.002	0.81

*Age at Diagnosis with SBC

Results from multivariate Cox regression analysis investigating factors associated with Five, 10, and 20-year overall survival. Specific comparisons between groups within categorical variables are listed in parentheses within the Variable column

### Univariate Analysis

Survival analysis was conducted using log-rank tests for comparison with univariate analysis and subanalysis of various factors. The results of the analysis for each variable are detailed below, while a summary of the results can be found in Table 4.

**Table 4:** Univariate analysis of overall survival among patients with chordoma of the skull base (SBC)

Variable	Five-year log-rank p-value	10-year log-rank p-value	20-year log-rank p-value
Age*	< 0.001*	< 0.001*	< 0.001*
Sex	0.958	0.426	0.358
Race	0.805	0.537	0.257
Marital Status (Married vs Single)	0.035*	0.222	0.294
Marital Status Subgroups	< 0.001*	< 0.001*	< 0.001*
Histological Type	0.478	0.456	0.681
Stage	0.017*	0.019*	0.005*
Chemotherapy	0.032*	0.008*	0.011*
Surgical Procedure (GTR vs None)	< 0.001*	< 0.001*	< 0.001*
Treatment Plan (surg + rad vs None)	< 0.001*	< 0.001*	< 0.001*
Surgery Performed	< 0.001*	< 0.001*	< 0.001*

^*^Denotes *p* < 0.05

This table presents the results of univariate analysis investigating the impact of various factors on OS among patients diagnosed with SBC. The variable sex consisted of male and female categories, race consisted of American Indian/Alaska Native, Asian/Pacific Islander, Black, White, and Unknown. Marital status was available coded as married vs single, and also with more detail categories: divorced, married (including common law), separated, single (never married), unknown, and unmarried/domestic partner. Analysis conducted with respect to both marital status coding methods in SEER. GTR: Gross Total Resection; surg + rad: surgery with radiotherapy, 99.4% had adjuvant RT

### Age

**Fig. 1:**
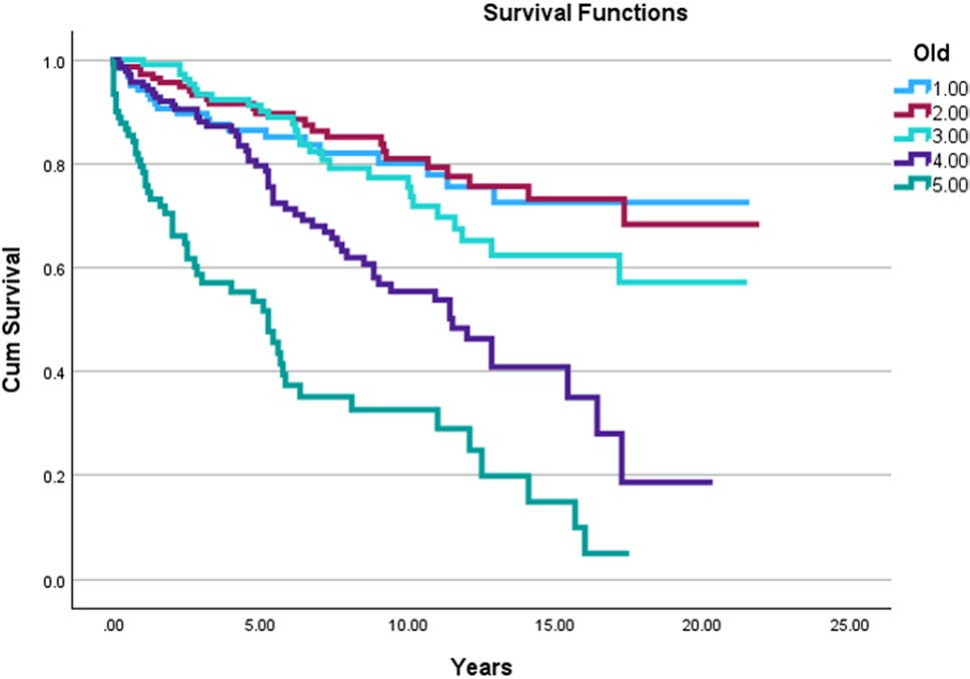
Kaplan–Meier curves depicting overall survival for SBC patients stratified by age at diagnosis in quintiles, demonstrating a significant difference in survival outcomes across all time points (*p* < 0.001 for each). **A**: Five-year OS curves, highlighting a notable disparity between the oldest (70–85 + years) and youngest (1–29 years) age groups, with respective OS rates of 60.4% [50.4%-70.5%] and 87.8% [82.0%-93.6%]. **B**: 10-year OS curves, further emphasizing the age-related survival gap, with OS rates of 49.5% [39.18%-59.72%] for the 70–85 + group and 84.6% [78.17%-90.94%] for the 1–29 group. **C**: 20-year OS curves, reinforcing the consistent trend of decreased survival with increasing age, with the 70–85 + group exhibiting a 42.9% [32.69%-53.02%] OS compared to the 82.1% [75.3%-88.9%] OS observed in the 1–29 group

Univariate analysis based on five groups, with each group being composed of ~ 20% of the entire cohort in this study (n = 630) revealed a significant difference in survival based on age at diagnosis across all survival timeframes: five-year (*p* < 0.001), 10-year (*p* < 0.001), and 20-year (*p* < 0.001; see Fig. 1). The 70–85 + age group exhibited notably lower survival rates compared to the 1–29 years age group. For the 70–85 + age group, the five-year overall survival (OS) was 60.4% [50.4%-70.5%], 10-year OS was 49.5% [39.18%-59.72%], and 20-year OS was 42.9% [32.69%-53.02%]. In contrast, the 1–29 years age group demonstrated significantly higher survival rates with a five-year OS of 87.8% [82.0%-93.6%], 10-year OS of 84.6% [78.17%-90.94%], and 20-year OS of 82.1% [75.3%-88.9%].

### Sex

No significant difference in overall survival was observed between males and females at any time point. The five-year OS for males was 84.5% [80.6%-88.4%] compared to 84.4% [80.3%-88.5%] for females (*p* = 0.958). Similarly, at the 10-year mark, the OS for males was 74.9% [70.3%-79.6%] and 77.6% [72.9%-82.4%] for females (*p* = 0.426). The 20-year OS also showed no significant difference, with males at 69.3% [64.3%-74.2%] and females at 73.2% [68.2%-78.3%] (*p* = 0.358).

### Race

Univariate analysis revealed no significant difference in survival based on race across all time points: five-year (*p* = 0.805), 10-year (*p* = 0.537), and 20-year (*p* = 0.257). Survival rates varied among racial categories. For American Indian/Alaska Native individuals, the five-, 10-, and 20-year overall survival (OS) was the same at 100.00% OS [100.00%-100.00%]. Asian or Pacific Islander individuals exhibited a five-year OS of 85.40% [77.72%-93.02%], a 10-year OS of 78.0% [69.1%-87.0%], and a 20-year OS of 74.4% [64.9%-83.8%]. Black individuals showed a five-year OS of 87.10% [75.30%-98.90%], and the same 10-, and 20-year OS of 83.90% [70.92%-96.82%]. Lastly, White individuals had a five-year OS of 83.90% [80.66%-87.06%], a 10-year OS of 75.00% [71.23%-78.77%], and a 20-year OS of 69.30% [65.28%-73.30%]. Overall, the OS percentages were consistent with the respective racial subcategories for each time point.

### Marital Status

**Fig. 2:**
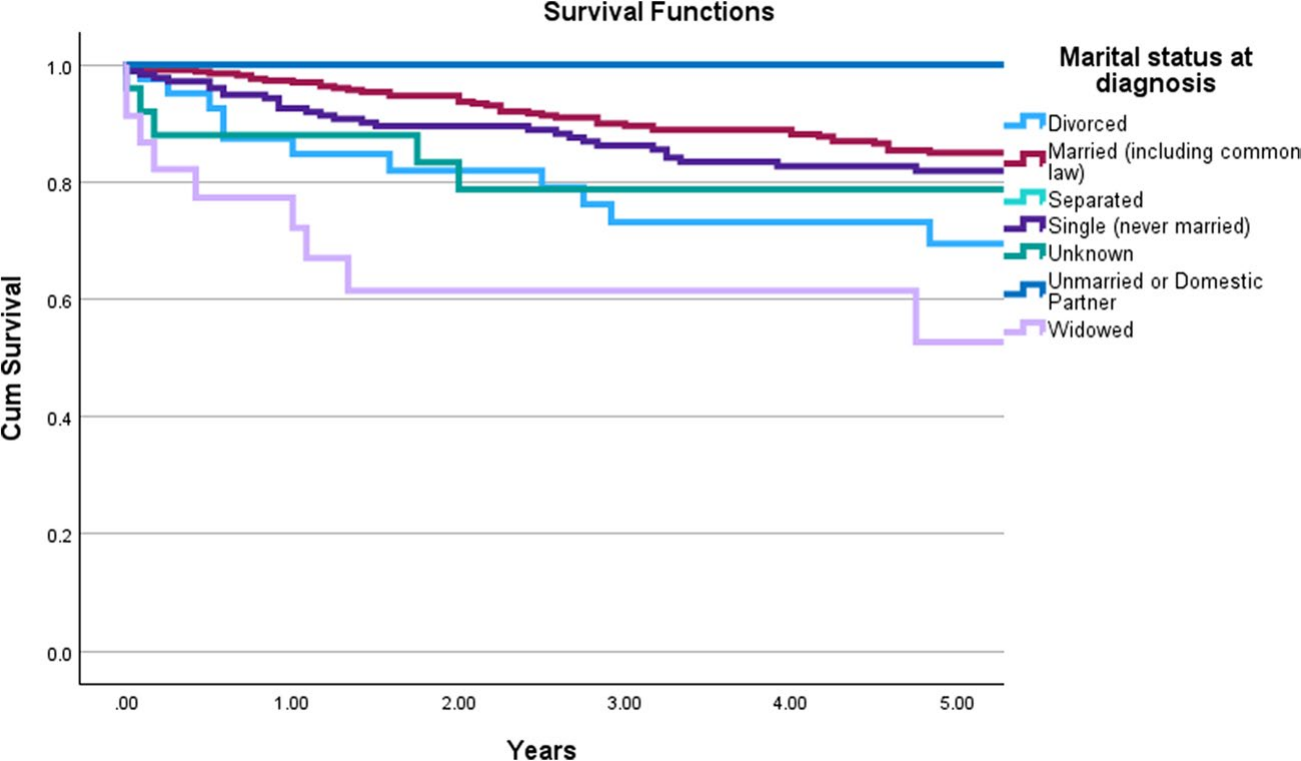
Kaplan–Meier curves depicting OS for SBC patients stratified by marital status at diagnosis. **A**: Comparison of survival curves for unknown, single, and married statuses, revealing a statistically significant difference (*p* = 0.035). Notably, at the five-year mark, married patients exhibited the highest mean OS of 87.2% [82.6%-91.8%], followed by single patients at 82.8% [77.8%-87.8%] and unknown at 76.2% [68.8%-83.6%]. **B**: Sub-analysis survival curves across various marital statuses, including divorced, married (including common law), separated, single (never married), unknown, unmarried or domestic partner, and widowed. The overall comparison demonstrated a statistically significant difference among the groups (*p* < 0.001). Survival analysis revealed that widowed patients consistently exhibited the lowest overall survival rates: 60.9% [40.9%-80.8%] at five years, 43.5% [23.2%-63.7%] at 10 years, and 34.8% [15.3%-34.8%] at 20 years. In contrast, unmarried or domestic partners, single (never married), and married patients demonstrated generally higher survival rates across all time points. For instance, at the 20-year mark, unmarried or domestic partners had 100% OS, single (never married) had 74.4% [68.1%-80.8%] and married had 71.8% [67.1%-76.7%] OS

Subanalysis of marital status revealed a complex relationship with survival. While there was no significant difference in 10-year (*p* = 0.222) and 20-year (*p* = 0.294) overall survival between married and single patients, a statistically significant difference was observed at the five-year mark (*p* = 0.035). Specifically, married patients had a five-year OS of 87.2% [83.7%-90.7%], while single patients had a lower five-year OS of 80.5% [75.6%-85.4%]. Figure 2 demonstrates these trends.

Further subanalysis of marital status, categorized into divorced, married (including common law), separated, single (never married), unknown, unmarried or domestic partner, and widowed, revealed a complex relationship with survival. While there was a statistically significant difference across all time points (*p* < 0.001 for each), the impact varied. Widowed patients consistently exhibited the lowest overall survival rates: 60.9% [40.9%-80.8%] at five years, 43.5% [23.2%-63.7%] at 10 years, and 34.8% [15.3%-34.8%] at 20 years. In contrast, unmarried or domestic partners, single (never married), and married patients demonstrated generally higher survival rates across all time points. For instance, at the 20-year mark, unmarried or domestic partners had 100% OS, single (never married) had 74.4% [68.1%-80.8%] and married had 71.8% [67.1%-76.7%] OS. These findings suggest an association between marital status and survival outcomes in SBC patients, with widowed status being a significant risk factor for poorer survival.

### Histological Type

No statistically significant difference in survival was found based on histological type during any survival timeframe. The three histologic ICD-O-3 codes identified in this analysis were 9370 (chordoma, not otherwise specified), 9371 (chondroid chordoma), and 9372 (dedifferentiated chordoma). The five-year OS for histological type 9370 was 84.3% [81.3%-87.3%], compared to 86.9% [78.4%-95.4%] for histological type 9371 (*p* = 0.478). Similarly, no significant differences were observed at the 10-year mark (9370: 76.0% [72.5%-79.5%], 9371: 78.7% [68.4%-89.0%]; *p* = 0.456) and the 20-year mark (9370: 71.4% [67.7%-75.1%], 9371: 68.9% [57.2%-80.5%]; *p* = 0.681). Histological type 9372 had a limited number of patients, and this was reflected in the survival analysis that was conducted: the OS for all three timeframes was the same, at 66.7% [13.3%-120.0%].

### Disease Stage

Univariate analysis demonstrated a significant difference in survival based on disease stage at diagnosis across all time points: five-year (*p* = 0.017), 10-year (*p* = 0.019), and 20-year (*p* = 0.005). Patients with local disease demonstrated a five-year OS of 89.20% [85.7%-92.7%], a 10-year OS of 82.60% [78.4%-86.9%], and a 20-year OS of 80.0% [75.5%-84.5%]. Individuals diagnosed with regional disease had a five-year OS of 81.70% [76.9%-86.5%], a 10-year OS of 72.40% [66.77%-77.95%], and a 20-year OS of 65.4% [59.5%-71.4%]. Lastly, the distant disease showed a five-year OS of 70.30% [55.55%-84.99%], a 10-year OS of 56.8% [40.8%-72.7%], and a 20-year OS of 48.60% [32.54%-64.74%].

### Chemotherapy

Patients receiving chemotherapy demonstrated a significantly decreased overall survival compared to those who did not receive chemotherapy or had unknown chemotherapy status (the SEER database codes this variable into two categories: Yes or No/Unknown). The five-year OS for patients receiving chemotherapy was 64.7% [42.0%-87.4%] compared to 85.0% [82.2%-87.8%] for those without chemotherapy or with unknown status (*p* = 0.032). This difference became more pronounced at the 10-year mark, with OS rates of 47.1% [23.3%-70.8%] for the chemotherapy group and 77.0% [73.7%-80.3%] for the non-chemotherapy/unknown group (*p* = 0.008). At the 20-year mark, the survival advantage persisted, with OS rates of 41.2% [17.8%-64.6%] for the chemotherapy group and 71.9% [68.4%-75.5%] for the non-chemotherapy/unknown group (*p* = 0.01).

### Surgical Procedure

A univariate analysis demonstrated a statistically significant and substantial survival advantage associated with surgical intervention versus no surgery at all time points evaluated (*p* < 0.001 for five-year, 10-year, and 20-year survival). Specifically, patients who underwent surgery exhibited a five-year overall survival (OS) rate of 87.3% [84.5%-90.1%], significantly higher than the 65.0% [54.5%-75.5%] observed in patients who did not receive surgery. This survival benefit persisted over time, with 10-year OS rates of 79.1% [75.7%-82.5%] for the surgery group and 56.3% [45.4%-67.1%] for the non-surgery group, and 20-year OS rates of 74.2% [70.5%-77.8%] and 50.0% [39.0%-61.0%] respectively.

Furthermore, a univariate subanalysis revealed a highly significant association between the extent of surgical procedure and overall survival across all survival timeframes.Patients who did not undergo any surgical resection exhibited the lowest survival rates, with a five-year OS of 64.60% [54.01–75.11], a 10-year OS of 55.70% [44.74–66.66], and a 20-year OS of 49.40% [38.34–60.39]. Individuals who underwent subtotal resection demonstrated improved survival compared to the those that did not receive any surgical treatment, with a five-year OS of 88.10% [84.73–91.34], a 10-year OS of 78.30% [74.11–82.52], and a 20-year OS of 74.00% [69.51–78.46]. Patients that underwent gross total resection showed even more favorable survival outcomes, with a five-year OS of 84.70% [79.13–90.19], a 10-year OS of 80.40% [74.21–86.47], and a 20-year OS of 76.10% [69.54–82.63]. Notably, patients with a surgical procedure coded as not otherwise specified or unknown displayed the highest survival rates among all groups, with a five-year OS of 94.70% [84.69–104.78], a 10-year OS of 84.20% [67.81–100.61], and a 20-year OS of 63.20% [41.47–84.85].

### Multimodal Treatment

Analysis of treatment plans revealed statistically significant differences in overall survival at five, 10, and 20 years, with all p-values < 0.001. Patients who received no treatment (None Group) exhibited low survival rates, with a five-year OS of 68.90% [57.23%-80.47%], a 10-year OS of 60.70% [48.39%-72.92%], and a 20-year OS of 54.10% [41.53%-66.60%]. Those who underwent surgery without RT showed improved survival compared to the those that received no treatment, with a five-year OS of 83.70% [78.77%-88.66%], a 10-year OS of 76.30% [70.59%-81.96%], and a 20-year OS of 69.80% [63.63%-75.90%].

Patients treated with RT only had the lowest survival rates with a five-year OS of 52.60% [30.18%-75.08%], a 10-year OS of 42.10% [19.90%-64.31%], and a 20-year OS of 36.80% [15.15%-58.53%]. Notably, patients that received both surgery and RT had the highest survival rates among all treatment plans, with a five-year OS of 89.60% [86.28%-92.83%], a 10-year OS of 80.90% [76.69%-85.10%], and a 20-year OS of 77.00% [72.51%-81.52%]. These findings strongly suggest that a combination of surgery and radiation therapy yields the most favorable long-term survival outcomes for patients.

## Discussion

This analysis of 630 SBC patients from the SEER database contributes to the ongoing discussion regarding the management and prognosis of this rare cancer. Our findings align with existing literature by demonstrating the significant impact of age and treatment modality on OS. Notably, older age at diagnosis (> 49 years compared to ≤ 49 years) was consistently associated with decreased OS at five, 10, and 20 years (HR = 2.54, 3.06, and 3.30, respectively; *p* < 0.001 for all), similar to observations by Teng et al. and Bohman et al. with age to be a strong predictor of poorer outcomes [2, 10]. Additionally, univariate subanalysis demonstrated significantly lower OS for patients in the 0–4 years age group compared to older age groups at all time points (five-, 10-, and 20-year OS), with the 0–4 years group having the second lowest OS of 24.0%, with only the 85 + years age group having lower survival. This result establishes significance when compared to a similar insignificant finding by Chambers et al., in which patients under ten years of age exhibited worse but not statistically significant survival (HR = 1.63, *p* = 0.22; [3]). Lee et al. similarly observed that patients between 0–29 years of age had a poorer chordoma-specific survival initially, but showed improved survival reported in Kaplan–Meier analysis if they survived the first 100 months [7]. This study is the first study to establish significance in the 1–4 years age group with a significantly worsened 10-year OS than every older age group besides 85 + years.

Several other unique aspects of our study enhance its contribution to the field. First, we examined survival trends across three distinct time points (five, 10, and 20 years), offering a more holistic understanding of long-term outcomes compared to all previous SEER-based analyses that often focused solely on three-, five-, and 10-year survival. For instance, Chambers et al. reported a five- and 10-year OS rate of 73.5% and 58.7% for cranial chordomas respectively, comparable to our rates of 84.4% and 76.2% [3]. By extending the analysis to 20 years, we provide valuable insights into the long-term trajectory of the disease, finding a 20-year OS of 71.1%, higher than the reported 10-year survival. We believe assessing the 20-year OS is particularly useful for chordomas given the relatively high reported survival rates in the literature. It is possible, given that the data in our analysis is more recent compared to other studies, that the increased OS seen could be the result of advances in treatment.

Second, our subanalysis of marital status revealed a compelling association between being widowed and decreased survival across all time points (*p* < 0.001 for each). This previously unexplored factor underscores the potential influence of social support systems on the long-term outcomes of SBC patients, potentially impacting post-treatment care, quality of life, and ultimately survival. Similar findings have been reported to both head and neck and brain tumors [8, 9, 12], though an analysis specific to chordomas is limited.

Finally, this study incorporated analysis of various treatment permutations, including combinations of surgery and radiation, providing a more comprehensive overview of treatment modalities compared to studies with simpler classifications. For instance, while Teng et al. found no significant difference in OS between gross total resection and subtotal resection [10], while our analysis of combined treatment modalities demonstrated a significant survival advantage for patients receiving both surgery and radiation compared to either unimodal or no intervention (five-year OS: 89.6% vs. 68.9%, *p* < 0.001). This is the first multicenter study to reveal significance associated with surgery and radiation used together, resulting in increased survival.

Our findings regarding the survival benefits of surgery (five-year OS: 87.3% with surgery vs. 65.0% without surgery, *p* < 0.001) are consistent with previous research emphasizing the importance of surgical intervention in SBC management [4, 6]. Our data comparing survival and specific surgical procedures (e.g., gross total resection vs. subtotal resection) supports studies reporting improved outcomes with gross total resection. Similarly, the observed trend of decreased survival with radiation therapy between all groups (10-year OS: 42.1% [19.9%-64.3%] for radiation only vs. 60.7% [48.4%- 72.9%] for neither surgery or radiation, *p* < 0.001) corroborates findings from previous SEER analyses and highlights the complexities surrounding the role of radiation in SBC treatment [3, 6]. These integrated findings may reflect selection bias, as radiation is often employed for inoperable or advanced-stage tumors, leading to a poorer prognosis. Further research is needed to elucidate the optimal use of radiation therapy in SBC management, considering factors such as timing, modality, and tumor characteristics.

Our analysis revealed a significant negative prognostic impact of chemotherapy (five-year OS: 64.7% for chemotherapy vs. 85.0% for no chemotherapy/unknown, *p* = 0.032), consistent with the general consensus that chordomas are relatively insensitive to conventional chemotherapy [11]. This data emphasizes the need to explore novel therapeutic agents and targeted therapies for SBC, as current chemotherapeutic options offer limited benefit.

Our analysis was limited in some regards. This was a retrospective analysis and subject to the bias that come with such an analysis. Information on surgery, RT, or chemotherapy given in a palliative setting is not known. Furthermore, information on the morbidity associated with treatment and/or patient frailty is unknown, though the authors acknowledge this plays a role in the selection of which treatment modalities are used in the treatment of SBC. Subanalysis with respect to neoadjuvant vs adjuvant RT in combination with surgery was not possible, as only 0.6% of eligible patients received neoadjuvant RT compared to adjuvant RT. Information on the specific doses or agents used with treatment are not available through the SEER Program.

## Conclusion

Overall, our study reinforces the importance of a multidisciplinary approach to SBC management, considering age, disease stage, marital status, and permutations of treatment plan. While surgery remains the cornerstone of treatment, the role of adjuvant radiation therapy and the potential for emerging targeted therapies require further investigation. By integrating our findings with existing research and addressing the limitations of current data, this leads to more effective and individualized treatment strategies for this challenging disease. Further research with more granular data on the surgical extent, radiation details, and molecular characteristics is crucial to optimizing treatment strategies and improving the lives of SBC patients.

## Supplementary Information

Below is the link to the electronic supplementary material.

Supplementary file1 (DOCX 133 KB)

## Data Availability

The data and materials that supported the findings of this study are available from the corresponding author upon reasonable request. Original data are available at Surveillance Epidemiology and End Results (SEER) database (https://seer.cancer.gov/data/).
