# Correction to: Comparative overall survival analysis of chordomas of the base of the skull from the Surveillance, Epidemiology, and End Results (SEER) program between 2000 and 2020

**DOI:** 10.1007/s10143-025-03819-0

**Published:** 2025-09-16

**Authors:** Kevin E. Agner, Michael C. Larkins

**Affiliations:** 1 The Ohio State University College of Medicine, 370 W. 9Th Avenue, 43210 Columbus, OH USA; 2 East Carolina University Brody School of Medicine, 600 Moye Blvd, 27834 Greenville, NC USA; 3 Department of Emergency Medicine, Boonshoft School of Medicine at Wright State University, 2555 University Blvd, Fairborn, OH USA

**Correction to: Neurosurgical Review (2024) 47:683**

10.1007/s10143-024-02815-0

The authors regret that they missed to indicate the below disclaimer in the original published article.

It is now added as an article note in the original article.

Disclaimer: “The views and opinions expressed in this article/presentation are those of the author(s) and do not necessarily reflect official policy or position of the United States Air Force, Defense Health Agency, Department of Defense, or U.S. Government.”

The original article has been corrected.

The original article can be found at 10.1007/s10143-024-02815-0.

