# Supplementary material for: Comparative overall survival analysis of chordomas of the base of the skull from the Surveillance, Epidemiology, and End Results (SEER) program between 2000 and 2020
Source: Neurosurg Rev. 2024 Sep 25;47(1):683. doi: 10.1007/s10143-024-02815-0 (PMC11424729; doi:10.1007/s10143-024-02815-0)

**SUPPLEMENTARY INFORMATION:**

**Fig. 1** Histogram depicting the age distribution at diagnosis for patients with chordoma of the skull base in the US between 2000 and 2020


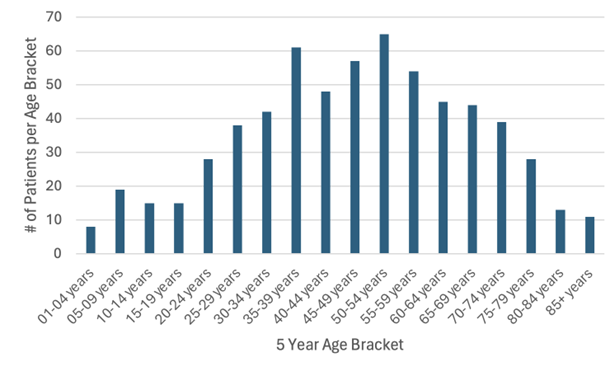


**Fig. 2** Kaplan-Meier curves depicting the 10-year OS for SBC patients stratified by age at diagnosis, demonstrating a significant difference in survival outcomes (p < 0.001). Younger patients consistently exhibit higher OS rates compared to older individuals. Notably, the 01-04 years age group has the second lowest 10-year OS of 24.0% [39.04%-61.0%], while the 10-14 years group demonstrates a much higher OS of 93.3% [80.79%-105.96%]. In contrast, the oldest age groups experience lower survival rates, with the 75-79 years group at fourth lowest OS of 50.0% [31.48%-68.52%] and the 85+ years group at a mere 18.2% [-4.61%-40.97%]. These findings highlight the oldest and youngest groups have substantially lower OS than a wide range of middle-aged groups


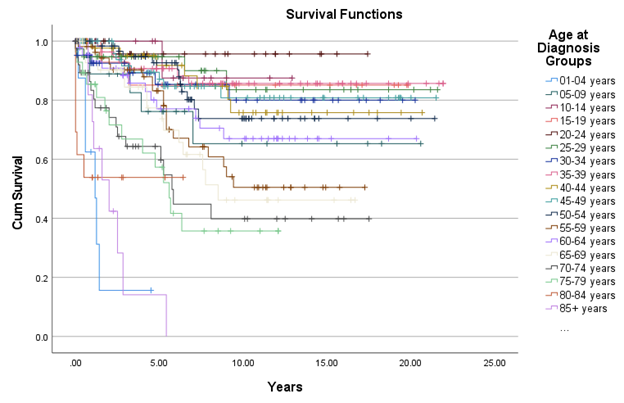

Supplement: Supplementary file 1 — Supplementary file1 (DOCX 133 KB) [file 10143_2024_2815_MOESM1_ESM.docx]
